# Knowledge and attitudes of thalassaemia among high-risk indigenous university students in Bangladesh: A pilot study

**DOI:** 10.1371/journal.pone.0287630

**Published:** 2023-07-07

**Authors:** Md. Mahbub Hasan, Khaza Md. Kapil Uddin, Syed Mohammad Lokman, Kallyan Chakma, Aung Chaing-U Pulu, Adnan Mannan, Enayetur Raheem, Shahed Ahmad Chowdhury, Mohammad Sorowar Hossain

**Affiliations:** 1 Department of Genetic Engineering and Biotechnology, University of Chittagong, Chattogram, Bangladesh; 2 Department of Emerging and Neglected Diseases, Biomedical Research Foundation, Dhaka, Bangladesh; 3 Department of Digital Health and Informatics, Biomedical Research Foundation, Dhaka, Bangladesh; 4 Chittagong Medical College, Chattogram, Bangladesh; 5 School of Environment and Life Sciences, Independent University, Dhaka, Bangladesh; UNITED KINGDOM

## Abstract

**Background and objectives:**

Thalassaemia is an inherited life-threatening but preventable haemoglobin disorder. South Asian countries, including Bangladesh, are the hotspots of the world’s thalassaemia belt. Indigenous communities are underprivileged and vulnerable to genetic disorders, including thalassaemia. Understanding the perspectives of thalassaemia of future community leaders (indigenous university students) is critical for developing a tailor-made preventive strategy relevant to their communities. In this study, we aimed to assess the level of knowledge and attitudes towards thalassaemia among indigenous university students and determine their thalassaemia carrier status.

**Methods:**

A cross-sectional survey was conducted among 251 tribal university students using a published questionnaire between May and October 2018. The main survey instrument consisted of 22 anonymous questions. Descriptive and inferential statistical procedures were used for data analysis.

**Results:**

More than half (55%) of the indigenous students had never heard the term ’thalassaemia’. Around half (49%) of the marriages in their communities were consanguineous. The mean knowledge score was abysmal (4.91±2.65 out of a 12-point scale), which was not associated with the consanguinity of their parent but home districts. Multiple linear regression of demographic variables on the total knowledge score revealed that the overall knowledge is significantly associated with their home district (p< 0.05). Participants from science disciplines scored more than 1 point than their counterparts from Arts and Humanities (p = 0.08615).

**Conclusions:**

For the first time, this study has identified knowledge gaps and misperceptions about thalassaemia among university students from indigenous communities in the southeastern region of Bangladesh. This study serves as a baseline for future interventions (premarital and prenatal screening) targeting future community leaders.

## Introduction

Thalassaemia is an inherited haemoglobin disorder. The only definitive cure for the disease is allogeneic hematopoietic cell transplantation which is costly, and there is a high risk of morbidity and mortality. However, thalassaemia is preventable. Once found to be localized in certain parts of the world, thalassaemia has become a global public health concern because of increasing international migration [1,2]. An estimated 1–5% of the world population are carriers of thalassaemia [2]. Nearly 90% of these births occur in developing countries [2]. In South Asia, particularly in India, Bangladesh, and Pakistan, thalassaemia has become a silent epidemic with an estimated 45–70 million thalassaemia carriers [3]. In Bangladesh, the prevalence of thalassaemia carriers among the general population (non-indigenous) is estimated to be around 6–12%, which translates into 10–19 million [4,5]. An estimated 60,000–70,000 patients have been suffering from severe forms of thalassaemia (β-thalassaemia major and HbE beta) in the country [3]. Despite a higher prevalence of thalassaemia carriers, people are mostly unfamiliar with this disease in Bangladesh [6].

The United Nations has declared that "indigenous peoples are recognized as among the world’s most vulnerable, disadvantaged and marginalized peoples" [7]. Out of approximately 390 million indigenous people globally, Bangladesh is the habitat of around 3 million people in these communities. Over one million indigenous people live in Bangladesh, which consists of 1.8% of the total population of Bangladesh (over 160 million). The indigenous or tribal people of Bangladesh mostly live in Chittagong hill tracts (CHT, 41%). Among them, Chakma, Marma, and Tripura comprise the largest ethnic minority group consisting of 90% of the indigenous population living in CHT [8,9]. Indigenous communities are underprivileged in education and have less access to healthcare services, mainly because their habitats are located in the country’s peripheral remote parts [10].

Moreover, the ratio of Pahari (mostly indigenous people living in CHT)-Bengali (migrants from other areas to CHT) is declining (1959: 90.39% to 1991:51.34%), and the dominance of Bengali culture also makes them isolated (name of several places changed) [11]. The concerted effect of all these social barriers is seen in the education of indigenous people. Nearly 82% of the children aged 5–19 years are enrolled in primary or secondary schools [12]. However, around two-thirds of them (65%) fail to complete primary schooling, and another 19% are dropped after completing primary education [12]. In contrast, the national school dropout rate reported by the Bangladesh Bureau of Statistics is 25% at the primary level and 22% at the secondary level [13]. The main barriers to keeping the tribal students in school are economic, social, remote access from the mainland, and armed conflicts among ethnic groups [12,14]. In addition, when students move out to different cities for higher education, they face social stigma due to their appearance, even from their peers [15].

Studies have shown that thalassaemia carriers (β-thalassaemia trait and HbE) are highly prevalent (39–49%) among indigenous populations [4,16,17]. However, the frequency of different thalassaemia types may vary from one region to another. About 70% of people from Asia, notably the northern regions of Thailand and Cambodia, carry the HbE variant. In contrast, the frequency of alpha thalassaemia in the Mediterranean regions, the Middle East to the Indian subcontinent, and East Asia range from 10–25% [18]. Many recent reports have described the higher prevalence of alpha and HbE than β-thalassaemia among tribes/indigenous communities from China, Vietnam, Malaysia, and Thailand [19–24].

Notably, the screening and diagnosis of genetic disorders are costly, e.g., thalassaemia diagnosis costs about $10-$15 in Bangladesh, and it is still facing challenges to implement in countries with low and middle income due to high out-of-pocket expenditures to get healthcare services [25,26]. In Bangladesh, about 64.3% of total healthcare expenditure is borne by households as out-of-pocket expenditure [27]. Moreover, if we also combined the scenario of indigenous peoples living in the CHT in Bangladesh with the vulnerable socio-economic conditions, inadequate access to healthcare, poor health awareness, and intra-tribal marriage practice, the higher prevalence of thalassaemia is expected to accumulate defective genes within the population [10,12,14].

In addition to the long-term consequences, there is another aspect of health equity for this minority group. Since indigenous people have less access to education [10,13], and 90% of the reserved jobs for Class I and II government jobs in Bangladesh are still vacant [28], it is speculated that individuals who get an opportunity for higher studies at the university level are expected to lead their communities. Therefore, understanding the perspective of thalassaemia among indigenous university students could be a critical step toward developing a tailored, effective preventive strategy by involving this community. In this study, we aimed to assess the level of knowledge and attitudes towards thalassaemia among indigenous university students and determine their thalassaemia carrier status.

## Materials and methods

### Study setting

This cross-sectional study was conducted between September 2018 and April 2019 in the students’ residential settings (including university-run residential halls and private halls) at the University of Chittagong. Because of its geographic location where most indigenous communities live and because governmental policy promotes higher education, this University reserves seats for indigenous students in every department and institute of eight faculties under a special quota system [29]. Thus, the University of Chittagong is the most appropriate setting for studying health issues like thalassaemia in indigenous communities in Bangladesh.

### Sampling

Convenience sampling was used in this study. After collecting information on the living preference of the tribal students at the University of Chittagong by interviewing the tribal enumerators who participated in this study, we found that female students generally live in university-run residential halls. At the same time, males preferred to live in privately maintained residential halls adjacent to the university campus, popularly known as "Cottage." For sampling, a total of eight residential halls and cottages of tribal students, including three ladies’ halls out of 4 (one was closed at the time of the survey) were selected for the study. Moreover, one classroom from the Department of Pali (the language of the Tripiṭaka) was also included because approximately two-thirds of the students in this department were from indigenous communities. Interviews in residential halls were conducted weekly on weekdays between 7:00 pm and 10:00 pm to maximize the number of participants between May 2018 and October 2018.

**Fig 1:**
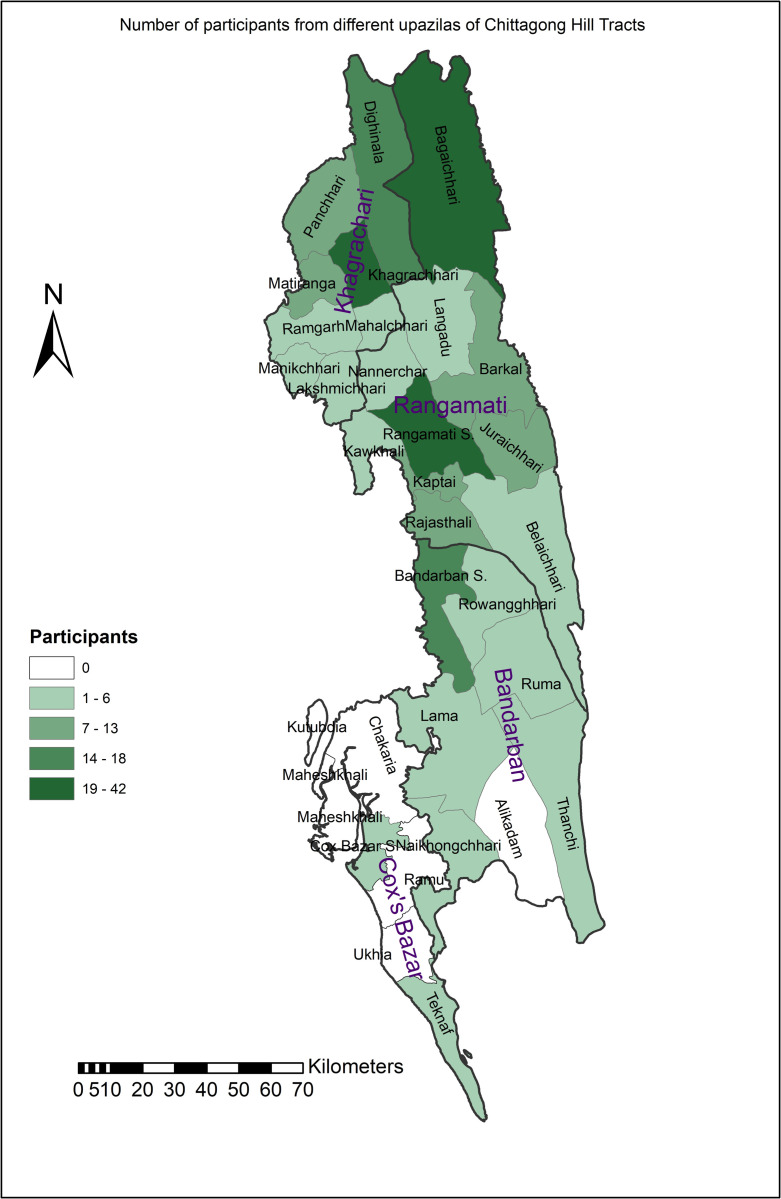
Spatial distribution of the tribal students participated in the survey of this study (N = 251).

In contrast, for the academic classroom, the time was 11:00 am and 12:00 pm. Only physically present students at each residential hall were enrolled in this study. For all cases, a general announcement was made through the appropriate authority (for university-run residential halls) that a survey on a health-related issue (not mentioning thalassaemia) would occur. On the survey day, a team of trained data enumerators, including the principal investigator, convened the students mentioned above’ residents. Students who agreed to participate without reservation were gathered in a large room of the respective residential hall (either a common room or prayer room). After signing the consent form, their responses were recorded in a printed questionnaire. The survey team supervised the participants during data capture. To avoid incidents of overlapping participation, it was announced that to refrain from participating in the study if they had already enrolled in another data collection site. This was confirmed by checking the demographic data of the questionnaire afterward. The geographical distribution of participants was carried out up to Upazila (sub-district level), and the spatial distribution was presented in Fig 1.

### Survey instrument

This study used a previously published questionnaire to assess college students’ knowledge and attitudes toward thalassaemia in Bangladesh [6]. In brief, the anonymous questionnaire consisted of 22 questions with two major sections prepared in the Bengali language to avoid the language barrier. The demographic section questionnaire was revised to capture the indigenous characteristics of the participants. One particular question was included in determining the consanguinity status by asking whether their parent is the first cousin.

Consent and demographic information of the participants were collected at the beginning of the survey. Participants were asked if they had heard of "thalassaemia". Those who answered "yes" were asked to proceed with the survey. The participant’s knowledge about thalassaemia was subsequently assessed on 12 items. Of these knowledge-related questions, nine items, Q1 to Q9 in Table 2, were recorded using a 5-point Likert scale (strongly agreed, agreed, do not know, disagreed, and strongly disagreed). These nine items were focused on general knowledge of thalassaemia, its carrier, and prevention. The responses were then converted into three groups, namely "Correct," "Incorrect" and "Don’t Know" to calculate the score on individual questions. The Q10 was open-ended, while the rest two were in Yes/No format. A correct response was given a score of one; an incorrect response and ’don’t know’ was scored as zero, with higher scores indicating better knowledge. Participants’ attitudes toward thalassaemia were assessed by eight items using a 5-point Likert scale. Data were managed and stored using REDCap electronic data capture tool hosted at Biomedical Research Foundation (BRF) for further processing (https://redcap.brfbd.org/). To assess the thalassaemia carrier status, a screening program was run using a standard protocol which is described in the supplementary methods.

### Statistical analyses

Descriptive and inferential statistical procedures were used to analyze the data. Categorical variables were presented using counts and percentages, and continuous variables were summarized using means and standard deviations. Pearson’s chi-square test with continuity correction was used when necessary to test the association between categorical variables. The Likert scale measurements were summarized by computing the total scores. Means and standard deviations were calculated for the scores. Association between demographic variables and total thalassaemia knowledge scores was calculated using multiple linear regression. We fit a multiple linear regression model assuming the knowledge scores as a continuous variable that may be predicted by the selected socio-demographic variables. In particular, we hypothesized that the level of education (year of study, whether 1st year, 2nd year, and so on) and the academic program they are currently enrolled in would be significant predictors. A p-value smaller than 0.05 was considered significant. All data were analyzed using SPSS Statistics for Windows, Version 25.0 (SPSS Inc, Chicago, Illinois).

### Ethics and consent

This study’s ethical approval was obtained from the Chittagong Medical College and Hospital (Memo: CMC/PG/2018/50, dated 17-04-2018). The data collection questionnaire did not contain any personal information of participants. Written consent was obtained from each participant.

## Results

### Respondent characteristics

A total of 251 university students from indigenous communities, about 55% of the total tribal students at the university of Chittagong, as per the admission office, participated in the study. Students came from 24 out of 25 Upazilas (sub-districts) of Chittagong Hill Tracts (consisting of Bandarban, Rangamati, and Khagrachari) except Alikadam of Bandarban (Fig 1). Most of them (91%) were followers of the Buddhist religion. Two-thirds of the participants were from Arts and Humanities disciplines, while the rest were from Social Science, Science, and Business Administration (Table 1).

**Table 1:** Demographic characteristics of participants and the proportion of the participants who heard the term/name of thalassaemia (N = 251).

Variables	All participants N = 251 n (%)	Have heard of thalassaemia n (%)		Pearson’s χ^2^	p
		Yes 112 (44.6%)	No 139 (55.4%)		
*Gender*					
Male	154 (61.4%)	62 (55.4%)	92 (66.2%)	3.068	0.080
Female	97 (38.6%)	50 (44.6%)	47 (33.8%)		
*Place of origin*					
Bandarban	32 (12.7%)	20 (17.9%)	12 (8.6%)	6.818	0.078
Cox’s Bazar	4 (1.6%)	3 (2.7%)	1 (0.7%)		
Khagrachari	94 (37.5%)	37 (33.0%)	57 (41.0%)		
Rangamati	121 (48.2%)	52 (46.4%)	69 (49.6%)		
*Discipline studying*					
Arts and Humanities	167 (66.5%)	71 (63.4%)	96 (69.1%)	16.33	0.001
Business Administration	21 (8.4%)	10 (8.9%)	11 (7.9%)		
Science	31 (12.4%)	23 (20.5%)	8 (5.8%)		
Social Sciences	32 (12.7%)	8 (7.1%)	24 (17.3%)		
*Year of study*					
1^st^ Year	26 (10.4%)	7 (6.3%)	19 (13.7%)	5.258	0.262
2^nd^ Year	81 (32.3%)	38 (33.9%)	43 (30.9%)		
3^rd^ Year	48 (19.1%)	19 (17.0%)	29 (20.9%)		
4^th^ Year	56 (22.3%)	27 (24.1%)	29 (20.9%)		
Masters	40 (15.9%)	21 (18.8%)	19 (13.7%)		
*Ethnicity*					
Chakma	164 (65.3%)	71 (63.4%)	93 (66.9%)	15.067	0.005
Marma	46 (18.3%)	24 (21.4%)	22 (15.8%)		
Tanchangya	16 (6.4%)	11 (9.8%)	5 (3.6%)		
Tripura	19 (7.6%)	2 (1.8%)	17 (12.2%)		
Others	6 (2.4%)	4 (3.6%)	2 (1.4%)		
*Religion*					
Buddhist	228 (90.8%)	109 (97.3%)	119 (85.6%)	10.218	0.001
Others	23 (9.2%)	3 (2.7%)	20 (14.4%)		

*Others: Different religious beliefs including Hindu, Cristian, Natural belief in god etc.

Among 251 participants, 123 (49%) reported that their parents were closely related to each other, second cousins, or closer (S1 Table). The rate of consanguineous marriage was significantly higher (p < 0.001) among tribes residing in Rangamati (63%), whereas the lowest was among tribals from Bandarban (16%). This practice was correlated with ethnicity (p < 0.001) and religious beliefs (p < 0.001), although most of the participants were followers of Buddhism, some of them believed in Hinduism, Christianity, etc. Moreover, Chakma students reported more (70%) parental consanguineous marriages (married to second cousins or closer).

More than half (55%) of the participants were unfamiliar with "thalassaemia." The proportion of students who had heard about thalassaemia was associated with study disciplines (p = 0.001), different indigenous ethnicity (p = 0.005), and religious belief (p = 0.001). While the majority (∼74%) of the students from the Science disciplines had heard of thalassaemia, ∼48% from Business Administration, ∼43% from Arts and Humanities and only ∼25% of students from Social Science faculty had heard of thalassaemia (Table 1).

### Knowledge on thalassaemia

Knowledge and attitude were assessed among 112 (44.6%) respondents who declared to have heard about "Thalassaemia" The frequently mentioned source of information about thalassaemia was the internet (28%), Friends (24%), and Facebook (22%) (S2 Table). Table 2 reports the distribution of responses to 12 knowledge-related questions among indigenous students who had heard about thalassaemia. Overall, respondents’ knowledge of the prevention and treatment of thalassaemia was poor. Only 22% answered correctly that thalassaemia is a preventable disease, and more than half of the participants replied that they had no idea about the prevention of the disease. The mean knowledge score was very poor (4.91±2.65 out of a 12-point scale) among the students who declared that they had heard about thalassaemia (S3 Table).

**Table 2:** Distribution of responses (correct/incorrect/doesn’t know) to 12 knowledge-related questions among students who have heard about thalassaemia (n = 112).

Questions	Correct n (%)	Incorrect n (%)	Don’t know n (%)
1. Thalassaemia is a contagious disease (disagreed and strongly disagreed)	62 (55.4%)	11 (9.8%)	39 (34.8%)
2. Thalassaemia is a genetic disease (agreed and strongly agreed)	71 (63.4%)	9 (8%)	32 (28.6%)
3. Thalassaemia could be transmitted through blood transfusion from a person with thalassaemia (disagreed and strongly disagreed)	19 (17%)	47 (42%)	46 (41.1%)
4. Marriage between two carriers can lead to a child with thalassaemia major (agreed and strongly agreed)	77 (68.8%)	3 (2.7%)	32 (28.6%)
5. If one parent is a carrier, the couple has a chance of having a child with thalassaemia disease (disagreed and strongly disagreed)	24 (21.4%)	56 (50%)	32 (28.6%)
6. Marriage between close relatives can increase the chance of thalassaemia (agreed and strongly agreed)	40 (35.7%)	22 (19.6%)	50 (44.6%)
7. Thalassaemia carriers are as healthy as normal people (agreed and strongly agreed)	39 (34.8%)	28 (25%)	45 (40.2%)
8. Thalassaemia is a preventable disease (agreed and strongly agreed)	22 (19.6%)	30 (26.8%)	60 (53.6%)
9. Thalassaemia is a completely curable disease (disagreed and strongly disagreed)	21 (18.8%)	32 (28.6%)	59 (52.6%)
10. Which part of the human body or organ is affected by Thalassaemia? (Blood or circulatory system)	25 (22.3%)	3 (2.7%)	84 (75%)
11. Anyone could be a thalassaemia carrier including you. (Yes)	67 (59.8%)	45 (40.2%)	NA
12. Thalassaemia can be identified by a blood test (Yes)	83 (74.1%)	29 (25.9%)	NA

Correct answers are provided in parentheses for each question.

Multiple linear regression of demographic variables on the total knowledge score revealed that the overall knowledge is significantly associated with their home district. The omnibus test showed that the model was overall significant with a residual standard error of 2.269 on 93 degrees of freedom, adjusted R-squared = 0.2704, and the F-statistic: 3.286 on 18 numerator degrees of freedom and 93 denominator degrees of freedom giving a p-value of < 0.001. Students from Khagrachari and Rangamati scored less than 2 points as compared to the reference district, Bandarban (Table 3). Moreover, students from science backgrounds scored more than 1 point than their counterparts from Arts and Humanities (p = 0.08615). However, knowledge scores were not associated with the consanguinity of their parents (p = 0.60605).

**Table 3:** Association between demographic variables and total thalassaemia knowledge scores among tribal students using multiple linear regression.

Variable	Estimate	Std. Error	*t*-value	*p-*value
(Intercept)	6.18384	1.43489	4.31	4.06E-05
*Gender*				
Female	Reference			
Male	0.48027	0.5112	0.939	0.34991
*Discipline*				
Arts and Humanities	Reference			
Business Administration	0.20785	0.82031	0.253	0.80053
Science (including Science, Engineering and Biological Sciences)	1.13661	0.65531	1.734	0.08615
Social Science (Including Law)	0.87089	0.93144	0.935	0.35221
*Year of study*				
1st Year	Reference			
2^nd^ Year	-0.13964	1.00746	-0.139	0.89006
3^rd^ Year	-0.29284	1.05711	-0.277	0.78238
4^th^ Year	-0.05991	1.05675	-0.057	0.95491
MS	0.22617	1.06718	0.212	0.83263
*Home district*				
Bandarban	Reference			
Cox’s Bazar	4.22751	2.76744	1.528	0.13001
Khagrachari	-2.23644	0.92362	-2.421	0.0174
Rangamati	-2.61268	0.92158	-2.835	0.00562
*Community types*				
Urban (District headquarter)	Reference			
Rural (Periphery)	0.24921	0.55314	0.451	0.65338
*Ethnicity*				
Chakma	Reference			
Marma	1.1648	0.78215	1.489	0.13981
Tanchangya	-0.65637	0.8801	-0.746	0.45767
Tripura	4.53444	2.92263	1.551	0.12418
Others	-3.13948	2.55196	-1.23	0.22172
*Religion*				
Buddhist	Reference			
Non-Buddhist	-4.04654	2.43856	-1.659	0.10041
*Consanguinity of parents*				
Non- Consanguineous	Reference			
Consanguineous	-0.29613	0.57224	-0.517	0.60605

### Attitudes towards thalassaemia

The attitudes of the indigenous students towards thalassaemia are presented in the S4 Table. The majority (91.1%) of the tribal students would prefer premarital screening to eradicate thalassaemia from society. More than two-thirds (69.6%) of them had positive attitudes toward donating blood to thalassemic patients. Three-fourths (75.9%) want to be a friend of thalassemic patients, and most (95.5%) showed positive attitudes to raising awareness among their community to prevent the disease.

### Prevalence of thalassaemia carrier among indigenous participants

A total of 114 participants underwent thalassaemia carrier screening, but the carrier status of four individuals could not be determined. We found that 48.2% of the participants were non-carriers, while 40.4% were carriers (38.6% E trait and 1.8% beta trait). E-disease (E-E genotype) was found among 7% of individuals, while one E-beta case (0.9%) was identified. Thalassaemia carriers’ carrier status was not significantly correlated with gender, ethnicity, or home district.

## Discussion

This study’s primary objective was to explore the knowledge and perceptions of thalassaemia among university students of the indigenous communities in Bangladesh. Against this backdrop, our study has identified a significant knowledge gap and misconceptions about thalassaemia among the indigenous communities’ highly educated segment.

Prior studies and our study found a higher prevalence of thalassaemia carriers among indigenous communities in Bangladesh. Consanguineous marriage practice might be responsible for the higher prevalence of thalassaemia carriers within the indigenous communities. Nearly 50% of marriages were consanguineous in the present study, while consanguineous marriage practice is less common in Teknaf, Chittagong region (17.6%) among non-indigenous populations [30]. Notably, around 70% of marriage practice was consanguinous in the Chakma tribe living in Rangamati. One reason behind the much higher prevalence of consanguineous marriage practice in Rangamati is that it is a district of lakes and hills where free movements are not accessible. In this study, 50% of the thalassaemia carriers were from Rangamati, indicating that the accumulation of defective genes might be linked to consanguineous marriage. Interestingly, there is no link between the total knowledge score and consanguinity of the parents of the participants (Table 3), which is expected as their access to education is poorer than the mainstream population.

We found that most tribal university students (55%) have not heard of the word- ’thalassaemia.’ Best of our knowledge, there is no word in the local dialect’ synonymous with thalassaemia in the indigenous communities.

On the other hand, the overall knowledge scores of the participants who heard about thalassaemia were poor irrespective of study disciplines and years of study at the university. For instance, only one-third (∼35%) of the respondents knew that thalassaemia carriers are as healthy as ordinary people. Only one out of five (20%) tribal students answered correctly that thalassaemia is a preventable disease. We have observed a notably lower score in knowledge among Chakma and participants from Rangamati (Tables S3 and 3); this might be due to the geolocation variation of Rangamati; this district harbours Kaptai Lake, which covers 10% of its total area, from other hill tracts regions which have a vast impact on peoples livelihood. In our previous study among college students on the mainland, we found that students with a science background were more familiar with thalassaemia since their school curriculum (standard IX and X) dedicated a few sentences to thalassaemia. Despite having thalassaemia-related information in the curriculum, college students’ overall knowledge scores were also poor [6]. In general, about 20% of high school students enroll in the science discipline, most of whom are from urban settings. In this study, we found that most indigenous students had arts and humanities backgrounds (66%), while only 12% had science backgrounds. As the first step for an effective thalassaemia prevention strategy, including thalassaemia-related information in all disciplines’ school curricula could be highly effective [6]. However, as mentioned earlier, nearly 85% of the tribal students dropped out before and after the primary level. Thus, unlike students in the general population, targeting school-level students might be ineffective for indigenous communities.

Given that indigenous communities are marginalized and live in the remote part of the country where there is less access to education, information, and healthcare facilities, without sensing the communities regarding genetic disorders like thalassaemia, any preventive attempt is expected to backfire. In this context, educating future community leaders (university students) about the consequence of thalassaemia could be a highly effective intervention approach to prevent thalassaemia in these communities.

### Limitations of the study

The questionnaire used in this study was not validated for the sampled population. However, a similar study was done recently nationwide and verified that they used 7 out of 10 knowledge testing instruments from our previous study. It is essential to mention that we assessed the knowledge level among the participants who heard of "thalassaemia" only; 55% of the participants who had not heard about the term were not included in the analysis. As such, it is difficult to verify how many of them took the term as a disease and explored it actively to understand the disease itself. Given the first baseline study in this highly vulnerable community, we relied on the tools we previously used, and part of that was validated by Alam NE, et al. 2022 [31]. Moreover, our cross-sectional study targeted mostly indigenous students of a particular region of Bangladesh (around half the tribal population live here).

## Conclusions

Best of our knowledge, this is the first study of its kind to identify knowledge gaps and misperceptions about thalassaemia among disadvantaged indigenous communities in the southeastern part of Bangladesh and perhaps elsewhere in the world. Our study has revealed that nearly half the participants had not heard about thalassaemia, and those who were familiar had a poor level of knowledge. Findings from this study would contribute to formulating effective intervention programs by engaging university graduates- the future community leaders of the most disadvantaged communities in the world.

## Supporting information

S1 Table Correlation of consanguineous marriage among families of tribal students of Chittagong University (N = 251). (DOCX)

S2 Table Major sources of information regarding Thalassaemia. (DOCX)

S3 Table Total thalassaemia knowledge scores of tribal students among different variables. (DOCX)

S4 Table Attitudes towards thalassaemia among students who have heard about thalassaemia (n = 112). (DOCX)

S1 Methods (DOCX)
