# Supplementary material for: Knowledge and attitudes of thalassaemia among high-risk indigenous university students in Bangladesh: A pilot study
Source: PLoS One. 2023 Jul 7;18(7):e0287630. doi: 10.1371/journal.pone.0287630 (PMC10328233; doi:10.1371/journal.pone.0287630)
Supplement: S1 Table — (DOCX) [file pone.0287630.s001.docx]

**Knowledge and attitudes of thalassaemia among high-risk indigenous university students in Bangladesh: A pilot study** Md. Mahbub Hasan^1,*^, Khaza Md. Kapil Uddin^1^, Syed Mohammad Lokman^1^, Adnan Mannan^1^, Enayetur Raheem^2^, Shahed Ahmad Chowdhury^3,4^ and Mohammad Sorowar Hossain^2,5,*^

1. Department of Genetic Engineering and Biotechnology, University of Chittagong, Chattogram 4331, Bangladesh.
2. Department of Emerging and Neglected Diseases, Biomedical Research Foundation, Dhaka 1230, Bangladesh.
3. Department of Digital Health and Informatics, Biomedical Research Foundation, Dhaka 1230, Bangladesh.
4. Chittagong Medical College, Chattogram 4203, Bangladesh.
5. School of Environment and Life Sciences, Independent University, Bangladesh.

* Corresponding author

[mahbub.hasan@cu.ac.bd](mailto:mahbub.hasan@cu.ac.bd) (Md. Mahbub Hasan); [sorowar.hossain@brfbd.org](mailto:sorowar.hossain@brfbd.org) (Mohammad Sorowar Hossain)

**S1 Table.** Correlation of consanguineous marriage among families of tribal students of Chittagong University (N=251)

| Variables | Parents are first cousine  n (%) | | Pearson’s χ^2^ | p |
| --- | --- | --- | --- | --- |
|  | Yes  123 (49%) | No  128 (51%) |  |  |
| *Permanent address* | | | | |
| Bandarban | 5 (4.1%) | 27 (21.1%) | 25.51 | < 0.0001 |
| Cox’s Bazar | 1 (0.8%) | 3 (2.3%) |  |  |
| Khagrachari | 41 (33.3%) | 53 (41.4%) |  |  |
| Rangamati | 76 (61.8%) | 45 (35.2%) |  |  |
| *Ethnicity* | | | | |
| Chakma | 115 (93.5%) | 49 (38.3%) | 85.803 | < 0.0001 |
| Marma | 4 (3.3%) | 42 (32.8%) |  |  |
| Tanchangya | 3 (2.4%) | 13 (10.2%) |  |  |
| Tripura | 0 | 19 (14.8%) |  |  |
| Others | 1 (0.8%) | 5 (3.9%) |  |  |
| *Religion* | | | | |
| Buddhist | 122 (99.2%) | 106 (82.8%) | 20.205 | < 0.0001 |
| Others* | 1 (0.8%) | 22 (17.2%) |  |  |

*Others: different religious beliefs including Hindu, Cristian, Natural belief in god etc.
