# Supplementary material for: Knowledge and attitudes of thalassaemia among high-risk indigenous university students in Bangladesh: A pilot study
Source: PLoS One. 2023 Jul 7;18(7):e0287630. doi: 10.1371/journal.pone.0287630 (PMC10328233; doi:10.1371/journal.pone.0287630)
Supplement: S1 Methods — (DOCX) [file pone.0287630.s005.docx]

**Knowledge and attitudes of thalassaemia among high-risk indigenous university students in Bangladesh: A pilot study** Md. Mahbub Hasan^1,*^, Khaza Md. Kapil Uddin^1^, Syed Mohammad Lokman^1^, Adnan Mannan^1^, Enayetur Raheem^2^, Shahed Ahmad Chowdhury^3,4^ and Mohammad Sorowar Hossain^2,5,*^

1. Department of Genetic Engineering and Biotechnology, University of Chittagong, Chattogram 4331, Bangladesh.
2. Department of Emerging and Neglected Diseases, Biomedical Research Foundation, Dhaka 1230, Bangladesh.
3. Department of Digital Health and Informatics, Biomedical Research Foundation, Dhaka 1230, Bangladesh.
4. Chittagong Medical College, Chattogram 4203, Bangladesh.
5. School of Environment and Life Sciences, Independent University, Bangladesh.

* Corresponding author

[mahbub.hasan@cu.ac.bd](mailto:mahbub.hasan@cu.ac.bd) (Md. Mahbub Hasan); [sorowar.hossain@brfbd.org](mailto:sorowar.hossain@brfbd.org) (Mohammad Sorowar Hossain)

**Supplementary methods:**

**Carrier screening and awareness campaign**

Blood samples were collected from interested participants on a convenient date in the Chittagong University Medical Centre for thalassaemia carrier screening. To maintain ethical guidelines, participants in the previous phase of the study were informed individually (texts and phone calls) to know who was interested to know their thalassaemia carrier status. Among them, 114 students (49 female and 65 male) participated in the second phase of the study and written informed consent was taken from each participant for collecting a 5ml blood sample. To protect the personal data a token number was generated against each participant to label the sample tubes and the personal data was stored securely in the REDCap system with restricted access to principal investigators only. Blood samples were then aliquoted into two Vacutainers containing K_3_EDTA (Ethylenediaminetetraacetic acid). Blood samples were collected while maintaining a cold chain and preserved in the laboratory refrigerator. Haemoglobin electrophoresis of preserved blood samples was carried out using Sebia full automated capillary electrophoresis system prescribed by the manufacturer (SEBIA - 27 rue Léonard de Vinci CP 8010 Lisses - 91008 Evry Cedex FRANCE). This technique separates different haemoglobin species in silica capillaries according to their electrophoretic mobility and electroosmotic flow at a high voltage in an alkaline buffer (pH 9.4). The result of the carrier status of participants was provided individually through an awareness program on thalassaemia organized in the presence of a haematologist. In this program, genetic counselling was included targeting those carrier-positive participants.
